# Artistic Value as a New Paradigm to Promote Ocean Conservation

**DOI:** 10.1007/s40656-025-00679-1

**Published:** 2025-07-02

**Authors:** Juliette Bessette, Thierry Pérez

**Affiliations:** 1 University of Lausanne, Anthropole, Lausanne, 1015 Switzerland; 2 Temps, Espaces, Langages, Europe Méridionale, Méditerrannée (TELEMME), Aix-Marseille Université, CNRS, Marseille, 13097 France; 3 Centre André Chastel, CNRS, Sorbonne Université, ministère de la Culture, Paris, 75002 France; 4 Institut Méditerranéen de Biodiversité et d’Écologie marine et continentale, Aix Marseille Université, CNRS, IRD, Avignon Université, Station Marine d’Endoume, Marseille, 13007 France

**Keywords:** Contemporary arts, Ocean conservation, Marine sciences, Ecosystem services, Arts & sciences

## Abstract

This position paper explores the artistic value that can be attributed to the ocean through works of art. The starting point is the economic argument used in ocean conservation, which is based on the concept of ecosystem services (ES), primarily considered as goods and services the ocean provides to society. Through an interdisciplinary approach, we briefly discuss the successes and limitations of this concept and the potential role of artistic value in relation to it. We then examine three case studies of contemporary artworks produced between 2015 and 2021, closely tied to marine natural sciences. Through these works, we explore how contemporary art can potentially operate within the ES framework, while also surpassing it through the specific type of value it induces in the ocean. Each of the case studies exemplifies a type of tangible engagement that art can provoke towards the ocean: (1) creating “embodied knowledge” of scientific understanding of the ocean (Nicolas Floc’h), (2) raising awareness through awe (Irene Kopelman), and (3) shaping a different type of concern for marine animals and their environments (Joan Jonas). Finally, through a critical analysis, we argue that a serious consideration of an artistic value attributed to the ocean can serve to place social points of reference that bridge canonical scientific knowledge with a diversity of other types of narratives related to it, thus contributing to a shift towards a new paradigm for supporting ocean conservation.

## Introduction

Over time, the quest to measure the value of marine ecosystems has gradually led us towards monetizing the ocean. This is the basis of the so-called “Blue Economy,” which also motivates all new national and international rules to protect the ocean. The economic (and sometimes commercial) evaluation of biodiversity emerged after the Millennium Ecosystem Assessment (MA, 2005) with aim of developing strategies to protect the environment. This laudable approach has been incorporated into European directives to define ecological responsibilities (1992/43*/*EEC, 2004/35/CE), and has led to the introduction of the ecological damage legal principle into national civil laws through environmental legislation. This principle aims to clarify and facilitate compensation for environmental damage, arguing that the degradation of an ecosystem and its services constitutes objective damage. This harm can be recognized in the case of environmental damage, and is subject to a qualified assessment under environmental law, thus opening up the possibility of such damage being brought before civil courts. It may then justify reparations, or, where appropriate, material or financial compensation. This legal approach suggests that damage caused to the environment can be repaired, reducing the concept of value to negotiations and compensations in a world currently driven by a willingness to destroy. Even if, just as when a piece of art is lost in a fire, we believe that the environment cannot be repaired in any way once it has been destroyed. In other words, if considered within a broader framework that defines the value of “nature” and ecosystems (Munns & Rea, 2017), the value attributed to marine ecosystems in this context is purely economic.

This economic value is associated with a utilitarian argument commonly employed within ocean science and policy communities, in a model for thinking of ocean conservation centered on the concept of ecosystem services (ES) – defined as “the functions and products of ecosystems that benefit humans, or yield welfare to society” (MA, 2005).While convenient to consider certain indicators, the use of this valuation framework has been questioned in numerous studies, underlying its weaknesses as the dominant approach to the environment-society relationship (Peterson, 2012; Lele et al., 2013; Schröter et al., 2014). The quest for alternative framing is very much alive.

In this paper, we argue this alternative framing must be considered in relation to sensibilities towards the ocean, and thus, more directly, through art. Art precisely reflects the evolving human sensibilities towards the ocean. In the 19th century global North, artists and writers contributed to its romantic view through works that embody an aesthetic of the sublime (an aesthetic that evokes awe and wonder, often in response to vast and overwhelming natural landscapes, emphasizing both beauty and terror). However, in the 20th century, they accompanied a shift towards a more scientific understanding of the ocean, coinciding with technological advancements that enabled exploration of the underwater world. Under these conditions, collaborations between marine biology researchers and artists have increased. Today, a comprehensive framework for general collaborations between arts and sciences has been theorized (Heylighen & Petrović, 2021), and there are more ocean-related collaborations than ever before. Unfortunately, these are generally limited to an analysis in terms of scientific mediation or entertainment. After a brief contextualization on ES framework, we examine three case studies that demonstrate how works of art, through their distinct means, offer singular ways to engage with the intricate challenges related to the oceanic environment and our relationship with marine life. We then discuss these artistic propositions collectively, within a “critical analysis” of the artistic value they confer to the ocean, which ultimately leads to shaping a new paradigm for supporting ocean conservation. In other words, we examine how this engagement through art can actually foster a deeper awareness of the ocean, thereby encouraging well-informed decisions and behaviors.

## Thinking about artistic value within and beyond the ES framework

If categorized within the ES framework, the artistic value of the ocean as defined in this paper – while often quite distant of the ES concept itself – would overlap with the so-called *cultural* services. This particular type of ES is arguably the most challenging to comprehend in terms of value, among the four recognized types. (1) The *provisioning* services are those whose economic value is easiest to measure, as they are characterized by all the “products” obtained from ecosystems^1^. The three remaining types are much harder to value, especially in the ocean. (2) The *regulating* services are defined as any benefit obtained from ecosystem functioning, e.g. climate regulation^2^. (3) The *supporting* services are those precisely related to ecosystem health, thus playing keystone roles in the maintenance of provisioning and regulating services^3^. (4) The *cultural* services are even more difficult to monitor and value as they include non-material benefits from ecosystems. For instance, spiritual enrichment in contact with the ocean is particularly strong in insular societies. On the other hand, recreational activities mainly take place in domesticated coastal waters, strongly impacted urbanized areas, or at the opposite, marine protected areas. Finally, the aesthetic values may eliminate the 90% of the ocean which is simply hidden from public view. We contend that art can play a special role by offering representational insight into parts of the ocean that remain invisible to the vast majority of humans. Further research would thus be required to more accurately determine the role of artistic value as a cultural ES, although it might carry the risk of perverse effect by assigning a monetary value to it from an anthropocentric perspective focused on human aesthetic experience^4^.

The debate in environmental ethics around the concept of ES notably focuses on the search for a non-anthropocentric valuation framework. The concept and the utilitarian standpoint it offers have indeed been criticized for their anthropocentric instrumental view of the environment, determining a value from the eye of the beholder (Munns & Rea, 2015). This is to the detriment of an ecocentric reasoning focused on the intrinsic values of nature or the environment (Auster et al., 2008; Schröter et al., 2014). It would nevertheless be erroneous to pit ecocentric and anthropocentric perspectives again each other, given that humans are one species within a complex socio-ecosystem. Instead, the aim should be to consider the ways in which these valuation frameworks overlap (Munns & Rea, 2017; Anderson et al., 2022; Himes et al., 2024). This article presents a conceptualization of artistic value as an extension of these discussions from the specific perspective of ocean-related human aesthetic experience. We intend to explore how contemporary art can intersect with both anthropocentric instrumental perspectives and considerations of the intrinsic value of the ocean, adding an extra argument based on its capacity to make tangible and concretely integrate different ways of engaging with the ocean.

### Nicolas Floc’h: “embodied knowledge” and conservation strategies

**Fig. 1:**
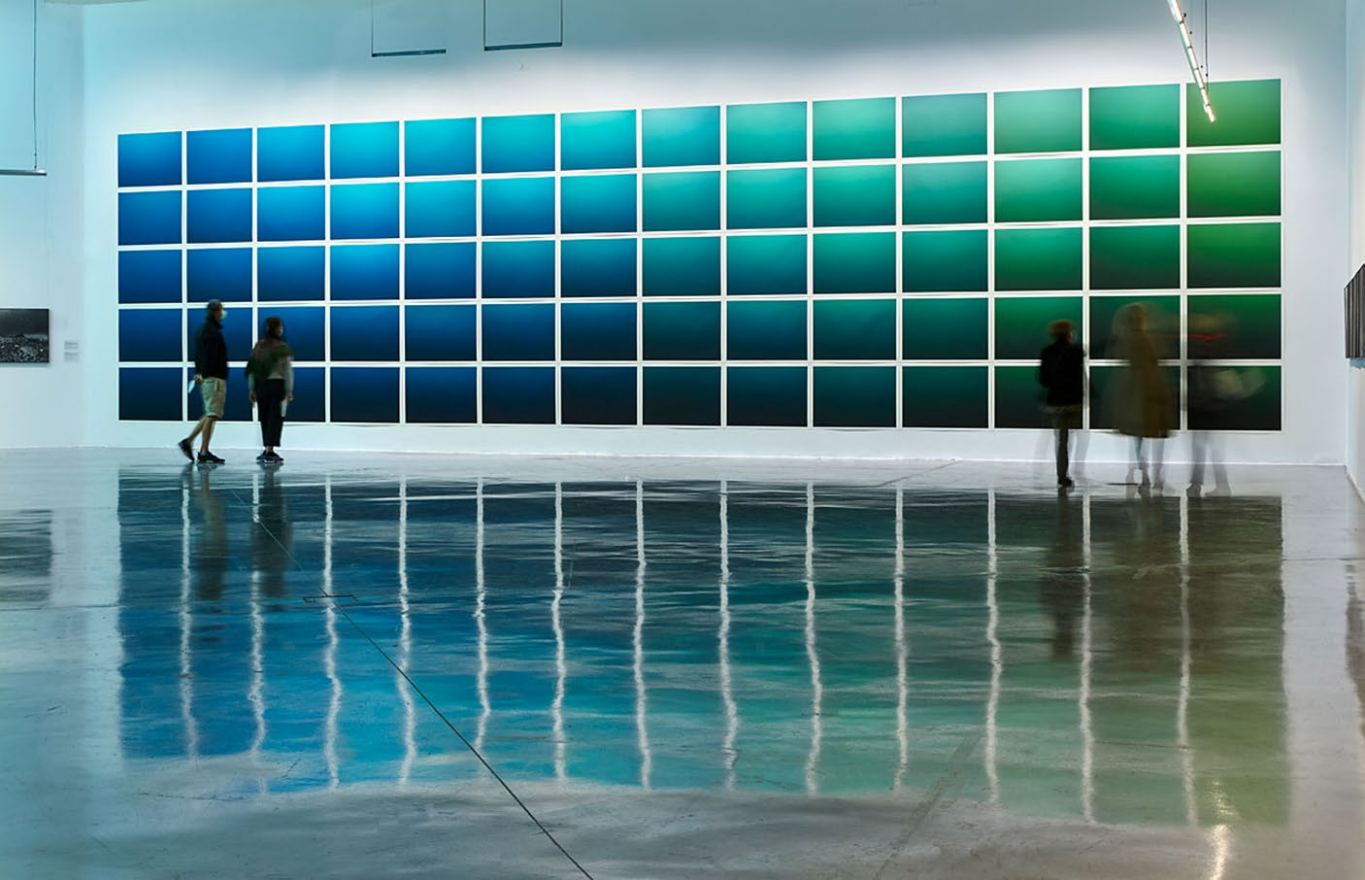
Nicolas Floc’h, *La couleur de l’eau*,* colonnes d’eau*,* de l’ile de Riou à la calanque de Cortiou (5 km)*, 70 photographs organized geographically, from the open sea to the coast, at depths ranging from- 5 to -30 m. France, 2019. Pigment prints, 72 × 101 cm each. *Paysages Productifs* exhibition, Frac Sud 2020 (Exhibition view: Laurent Lecat/ Frac Sud) © Nicolas Floc’h-Adagp, Paris, 2025

In the the philosophy of art, “embodied knowledge” is a concept used to denote the artistic capacity to contribute, through affect, to an understanding of the world that cannnot be achieved solely through the accumulation of scientific knowledge (Johnson, 2019)^5^. *The Color of Water* project (Fig. 1), by French artist Nicolas Floc’h, was launched in 2016 in collaboration with oceanographer Hubert Loisel from the Wimereux Marine Station on the French Opal Coast (northern France), itself named after the colors produced by the changing light on the water’s surface. However, for this series of photographs, the artist turned his attention to the underwater environment and how the concentration of suspended matter – both organic and inorganic – in the water column determines its color. In the ocean, phytoplankton is the main contributor to photosynthesis and primary production (Cloern et al., 2014), making it the first link in many food webs. The photosynthetic activity of phytoplankton is a determining factor in water color. In Floc’h’s photographs, the green range indicates the predominance of phytoplankton, while the blue range indicates less phytoplankton. As Sophie Blanchard puts it:

“In the scientific field, the primary data on water color is obtained from samples and satellite images. Nicolas Floc’h supplements this data by photographing the color inside the water mass using a precise protocol of double shots, one polarized and the other non-polarized. Polarization measurements are essential from a scientific standpoint, as they provide information on the chemical nature and size of suspended particles, including phytoplankton. Like an inventory, Nicolas Floc’h classifies and archives his photographs according to the geographical locations where they were taken. This approach allows him to document change of seascapes. The artist makes raw files of all his images, available to scientists, creating a photographic archive for research purposes^6^” (Blanchard, 2023).

This process can also illustrate the impact of human activities on the ocean. For example, the Mediterranean is known for its oligotrophy, which refers to very low average phytoplankton production. In a series of photographs taken in 2019 in the marine protected area of the Calanques National Park, near Marseille (South of France), the artist showcased abnormally high concentrations of phytoplankton in stretches of coastline. These areas were marked by a green color in the water, which was attributed to wastewater discharge. This wastewater from industrial, agricultural, and domestic activities is typically laden with organic matter and/or mineral elements that stimulate phytoplankton production and, in some cases, blooms of toxic microalgae. In this case, it can lead to disorders in the functioning of coastal ecosystems (Blanchard, 2023).

While the color of water has been a topic of concern in the field of ocean sciences since the 19th century and the intensification of scientific expeditions, it has recently taken on a particular dimension thanks to satellite tools and algorithmic processing in optical and biogeochemical measurements (Coble, 2007). The systematic and documentary method used by Floc’h through his direct photographs of the water mass results in the collection of additional data. This data, transmitted through pieces of art, can contribute to raising public awareness of the issue of coastal water composition. The artworks visual result is produced by the way the photographs are presented, in the form of a range of colors within large groups. Through a process of color comparison, we can perceive ecological dynamics that are invisible on the scale of individual underwater experience. At the same time, the works can be used to draw attention on scientific concepts such as bio-indication, considered the “most important biological method of environmental assessment” (Wiłkomirski, 2014). This approach has been widely integrated into monitoring programs, such as those in Europe where it was applied in accordance with the Water Framework Directive (2000/60/EC). Here, Floc’h produces representations of living things that are not focused on individualistic representations, as is typically the case in the history of art, but are rather anchored in ecosystemic dynamics. That way, his works can allow for the acquisition of “embodied knowledge”, serving as a way to engage with complex scientific information through an emotional connection.

**Fig. 2:**
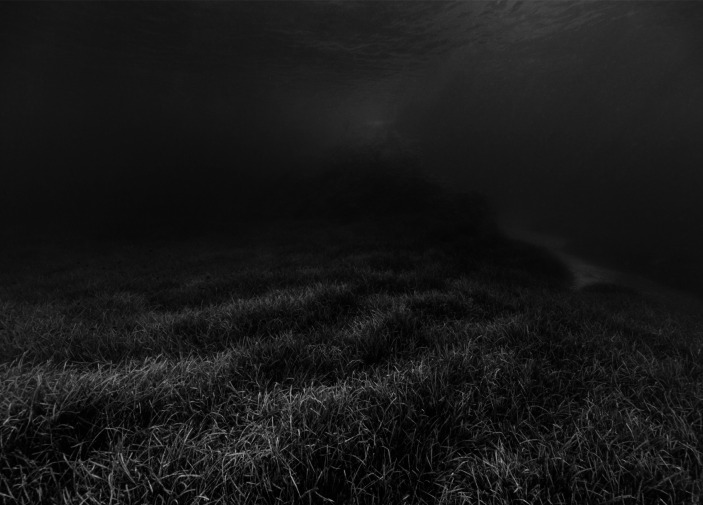
Nicolas Floc’h, *Invisible – Anse du Sec*, *-8 m*,* Posidonia meadows*,* rock*,* sand*, “Productive Seascapes” series, 2018–2020, black and white photograph, piezo on fine art paper, 72 × 101 cm © Nicolas Floc’h-Adagp, Paris, 2025

Flc’h also worked on the Calanques National Park territory in another series of photographs titled *Invisible* (2018–2020) (Fig. 2). In this series, he documented the condition of underwater “landscapes” along the Park’s 162 km of coastline and islands, mainly through snorkeling. He captured an image of the seabed every 6 m, resulting in a total of 30,000 shots. This project was carried out as part of a residency supported by the Park itself. The artist received support from the French Ministry of Culture as a public commission, marking it as the first of its kind dedicated to the underwater world. In addition, the photographic collection was intended to be used for scientific research at the OSU Institut Pythéas^7^, which was also behind the commission. It was meant to provide additional material for a different perspective on underwater seascapes that have been the subject of extensive scientific research over the past 60 years. “This is an unprecedented baseline that may allow scientists and managers of the National Park to record the development of our underwater landscapes in 10, 20, 30… years”, states the Park. The artistic project also included “50 small original prints to be displayed in publicly accessible structures within the communes of the National Park” (Calanques National Park, 2021); the images then to serve as support for the Park’s conservation initiatives. Directly following on from this project, in 2022 the Calanques National Park launched an environmental planning initiative in the form of France’s first “underwater landscape plan”. This initiative was funded by the French Ministry for Ecological Transition as a a management plan based on the human emotions that are evoked by the concepts of landscape/seascape and aimed to encourage local decision-makers, stakeholders, and residents to take responsibility for the underwater environment. The underwater space constitutes 93% of the Park’s territory, yet it remains largely unfamiliar to many. In an effort to “preserve natural spaces” in this area, the Park explains it aims to “develop a strategy and action plan that will be incorporated into the regulatory processes that impact the landscape” (Calanques National Park, 2022). The plan is therefore committed to conducting research, while also being fully integrated into environment management issues.

In this case study, we can refer to Floc’h as an official artist of initiatives that showcases a combination of art and science for ocean conservation or environmental management, in contexts that commonly use the ES framework^8^. His pieces, especially commissioned ones, are tied to these strategies when aiming to complement them by fostering embodied knowledge – transforming experience into understanding – and engaging diverse audiences. Notably, these artworks and the data they contains are also intended for scientific use. However, a dedicated study would be needed to assess their actual impact on scientists, as well as on the general public and policymakers. By conferring artistic value to the ocean, these pieces tend to offer a means to develop a personal, qualitative understanding of marine ecosystems, even without direct experience of the ocean. While rooted in the epistemic culture of marine sciences, Floc’h’s work diverges significantly from conventional scientific narratives, which often fail to reach and impact these audiences. Ironically, in the case of the *Invisible* series, the production of these artworks is linked to two French ministries in a state, while France’s conservation strategies are facing strong criticism – particularly regarding marine protected areas (Claudet et al., 2021; Bloom, 2022). This raises questions about the potential instrumentalization of art for the sole purpose of political communication, even as it holds great promise for fostering embodied knowledge.

### Irene Kopelman: raising awareness through awe

Argentinian artist Irene Kopelman first turned her attention to marine microorganisms in 2016 as part of a research project that also explored the color of water. This exploration sparked her interest in plankton. In 2019, she initiated a collaboration with two research laboratories specializing in life and health sciences, specifically focusing on marine invertebrates. For a period of 14 months, she was hosted in residence as part of the Côte d’Azur University’s advanced research program in the South of France. This collaboration involved the Institute for Research on Cancer and Aging - Nice (IRCAN) and the Villefranche-sur-Mer Developmental Biology Laboratory (LBDV)^9^. While there, the researchers, particularly Éric Röttinger and Stefano Tiozzo, introduced her to their ongoing research. The artist decided to focus her attention on two species of marine invertebrates, which were the respective study subjects of each laboratory: the “starlet” sea anemone *Nematostella vectensis* (IRCAN) and the colonial ascidian *Botryllus Schlosser*, also known as the star tunicate (LBDV). Both laboratories were investigating the regeneration process of these animals (Guenin, 2022). Regeneration can occur during their life cycle as a means of repairing lost tissues and preserving their genetic heritage, thus prolonging their lifespan under stressful conditions. These mechanisms, which can be classified as supporting services, therefore provide direct information on the resilience of these marine invertebrates. As the genes involved in these mechanisms are similar to those found in vertebrates, including humans, studying them in a laboratory setting can provide a model for understanding the cellular and molecular basis of regeneration and longevity in our own species. It can also shed light on resistance to age-related diseases, such as cancer (Röttinger, 2021).

**Fig. 3:**
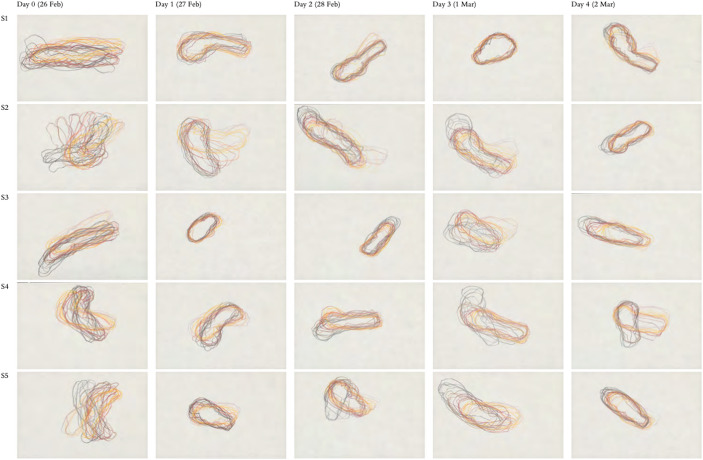
Irene Kopelman, *Nematostella Regeneration Experiment*, 2021, series of 50 drawings, colored pencil on paper, 19.5 × 29.8 cm each

**Fig. 4:**
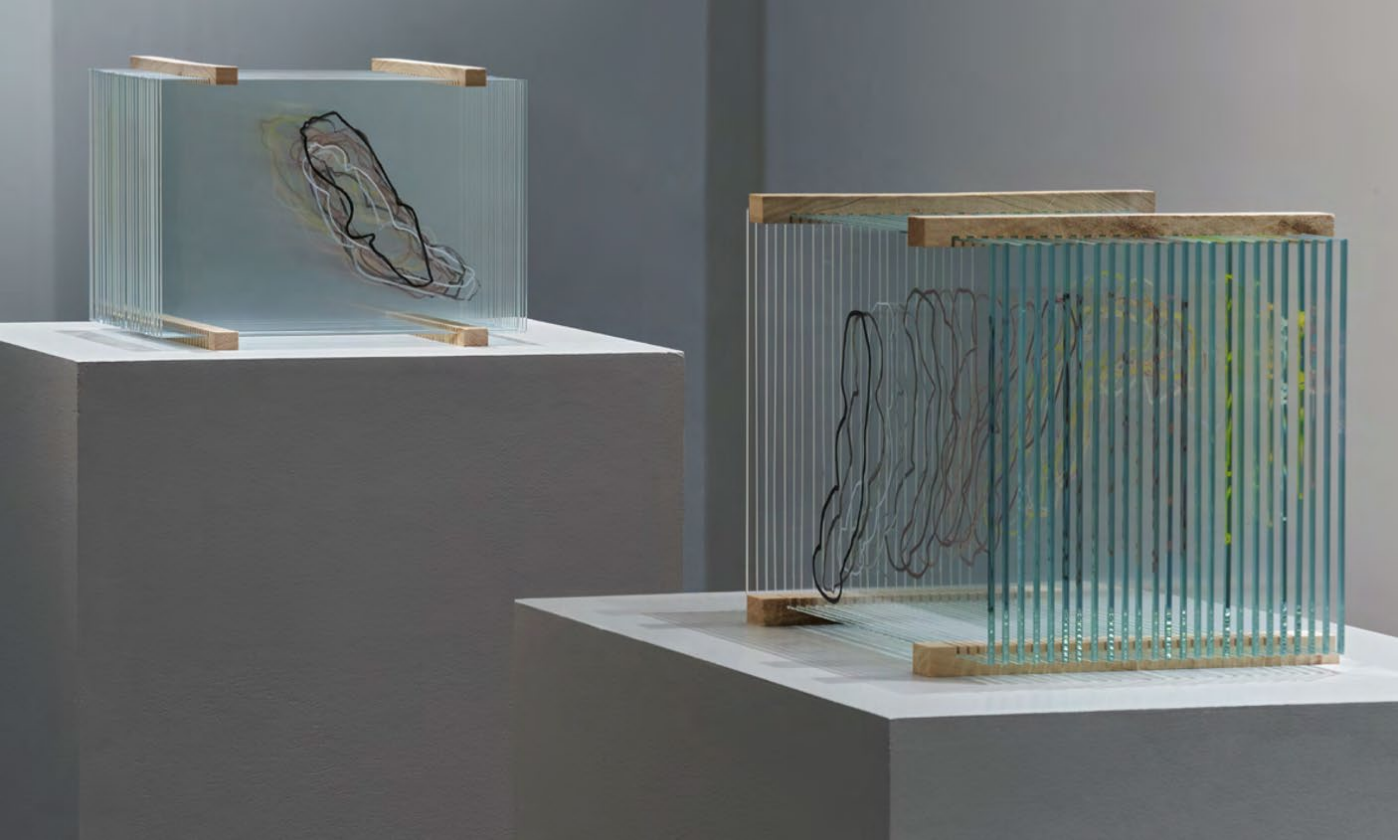
Irene Kopelman, *Nematostella in Motion*, 2021, series of 5, enamel on glass, 21 × 31.5 × 19 cm. © Thomas Lannes

However, during her residency, the artist chose not to use the genetic and genomic data collected by the researchers. Instead, she took a different approach establishing her own protocols to represent the regenerative capacity, growth phases, and changes in the properties and colors of these invertebrates. This led to a body of works titled *Marine Models. Drawing Regeneration* (2022–2023) (Figs. 3 and 4), in which she proposed a visual approach to regeneration through the use of color, movement, and texture. This work is nevertheless not alien to the research process of the laboratory teams, for whom it can provide a basis for scientific observation. “Irene observes and notes morphological details and color changes that are not part of our protocol. We could learn from these observations, as well as from behavioral traits that could provide new phenotypic markers”, explains Stefano Tiozzo (Tiozzo in Guenin, 2020).

This research residency culminated in Spring 2021 with workshops open to the general public at the MAMAC, the Nice Museum of Contemporary Art. Kopelman prepared for it by conducting months of observations on the animals, engaging in discussions with researchers, and consulting historical documentation on the animals’ regeneration capabilities (Kopelman, 2022). The aim of the workshops was to enable the public to actively engage with them, usually invisible to their eyes beneath the surface of the ocean, “experiencing the very (breathing) presence of the animals while working with them”, as the artist explains (Kopelman, 2021). To do so, *Nematostella vectensis* and *Botryllus schlosseri* were set up in aquaria at the center of the workshop installation, with their images projected onto the wall after being magnified under a microscope. Over two weeks of exhibition-workshops, they evolved and followed their life processes, including regeneration. For Kopelman, their physical presence at the center of the workshops was essential to the experience of encounter and “coexistence” between humans and these other species. As for their presence in the form of large-scale projected images, this was important for the public “both for objective observation and for visual stimulation (aesthetic qualities).” Laboratory biological models thus became models for artistic investigation in which “both aspects were equally important and complementary” (Kopelman, 2021, 2022).

**Fig. 5:**
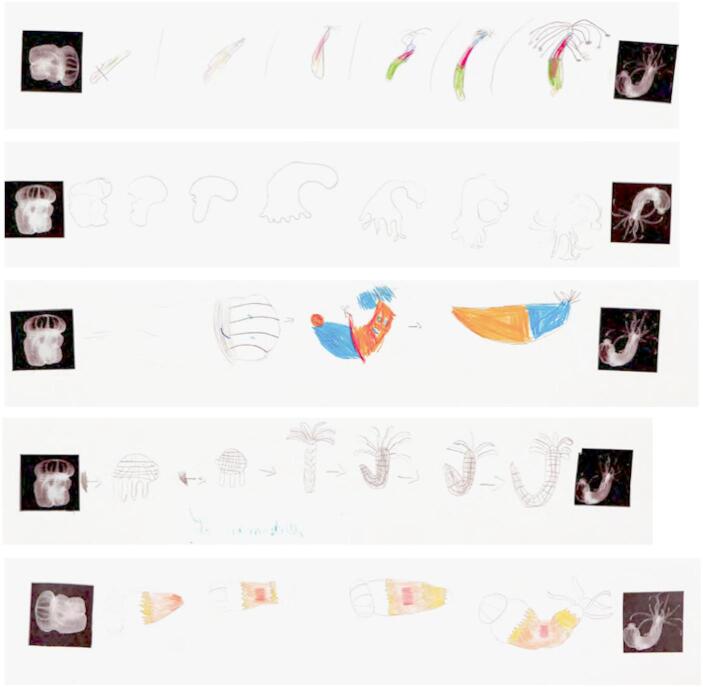
Workshop Drawings, *Frameline: Nemostella*. June 8–11, 2021 Participants: École Auber, École Bischoffsheim, Family workshop, Workshop for teenagers, École Bon Voyage Nice, École du Port, École Saint-Pierre de Féric. Assignment: “The *Nematostella* has been eaten by a fish or cut in the environment by accident, but a part of it remains. Draw stages of regeneration of *Nematostella* onto a paper frieze, starting with a remaining fragment and finishing with the reference image of the fully regenerated animal.”

Participants, especially children and adolescents, were invited to draw the two models in various scenarios involving a regeneration process. For instance, they were asked to depict a situation where one of the animals has lost a part of its body and launched the regeneration process (Fig. 5). “Children were free to use their imagination alongside scientific images as a reference”, explains the artist. For her, the practical exercise of drawing should not be limited to a formal level. Instead, it should be viewed as a way to deepen our understanding of the animals’ life cycle and of their ability to regenerate, taking into account the unique characteristics of each species. “Drawing is a way of thinking and processing of what we see through material and body activity. […] One model was colonial [the “starlet” sea anemone], the other was solitary [the star tunicate]. That itself posed a challenge for drawing – one model assumed/expanded in spatial patterns, whilst the other was enclosed” (Kopelman, 2021). Here, the process of drawing can be understood as a process of active acquisition of embodied knowledge. Above all, however, it is intended to evoke a sense of awe at the extraordinary process of regeneration, followed by admiration and respect for these marine forms of life that are removed from the everyday human life on earth. These workshops were designed to raise public awareness about the importance of preserving these animals and their ecosystems, regardless of their usefulness to humans. Kopelman also conducted workshops in various schools, and with scientists and their families.

In this case study, the artist chooses to distance herself from the scientific goal of human-oriented research conducted on the animals and from the broader context of valorisation and commercialisation of biotechnologies. In our view, this implicitly reflects her commitment to fostering a deeper relationship with marine species, one she hopes to cultivate within society. The artistic value of the ocean derived from this project stems from an observational approach to ocean life, which, while partly rooted in documentary aspects, focuses on highlighting the wonder of the regeneration process, with active involvement through drawing. The remarkable biological properties of the two species in question, which demonstrate the resilience of living organisms, have the ability to captivate the public’s attention and evoke a sense of awe, i.e. of deep admiration combined with astonishment. This sense of awe is partly caused by Kopelman’s idea of the co-presence of ocean species and the human species in the same location. But its activation mainly hinges on a sensitivity – whether developed or not – to the intrinsic value of these organisms and their life processes, which then takes precedence over the researchers’ more practical concerns. The artist is shifting away from a utilitarian approach to comprehend these lifeforms and the benefits they can provide to human societies within the ES framework – even if her art might partly be attributed to the cultural ES tied to the aesthetic pleasure that humans derive from observing and drawing these animals. Her approach aligns with the increasing focus on non-human agency in societies of the global North, distancing itself from the ES concept (Peterson, 2012). In this context, we start to encounter a type of artistic value that transcends any notion of ocean conservation driven solely by human benefit.

### Joan Jonas: conservation motivations through a different consideration of the ocean

The installation-performance *Moving Off the Land II* (2016–2020) by U.S. artist and poet Joan Jonas was first presented in 2019 at Ocean Space, a contemporary art space in Venice, Italy, dedicated to artistic work related to the ocean^10^. Since the late 1970s, Jonas (born 1939) has focused her artistic work on words, language, myths, and stories. In this piece, she has embraced the rich tradition of storytelling about the ocean and its inhabitants. The result is an installation that equally embraces various types of narratives that can be associated with them.

**Fig. 6:**
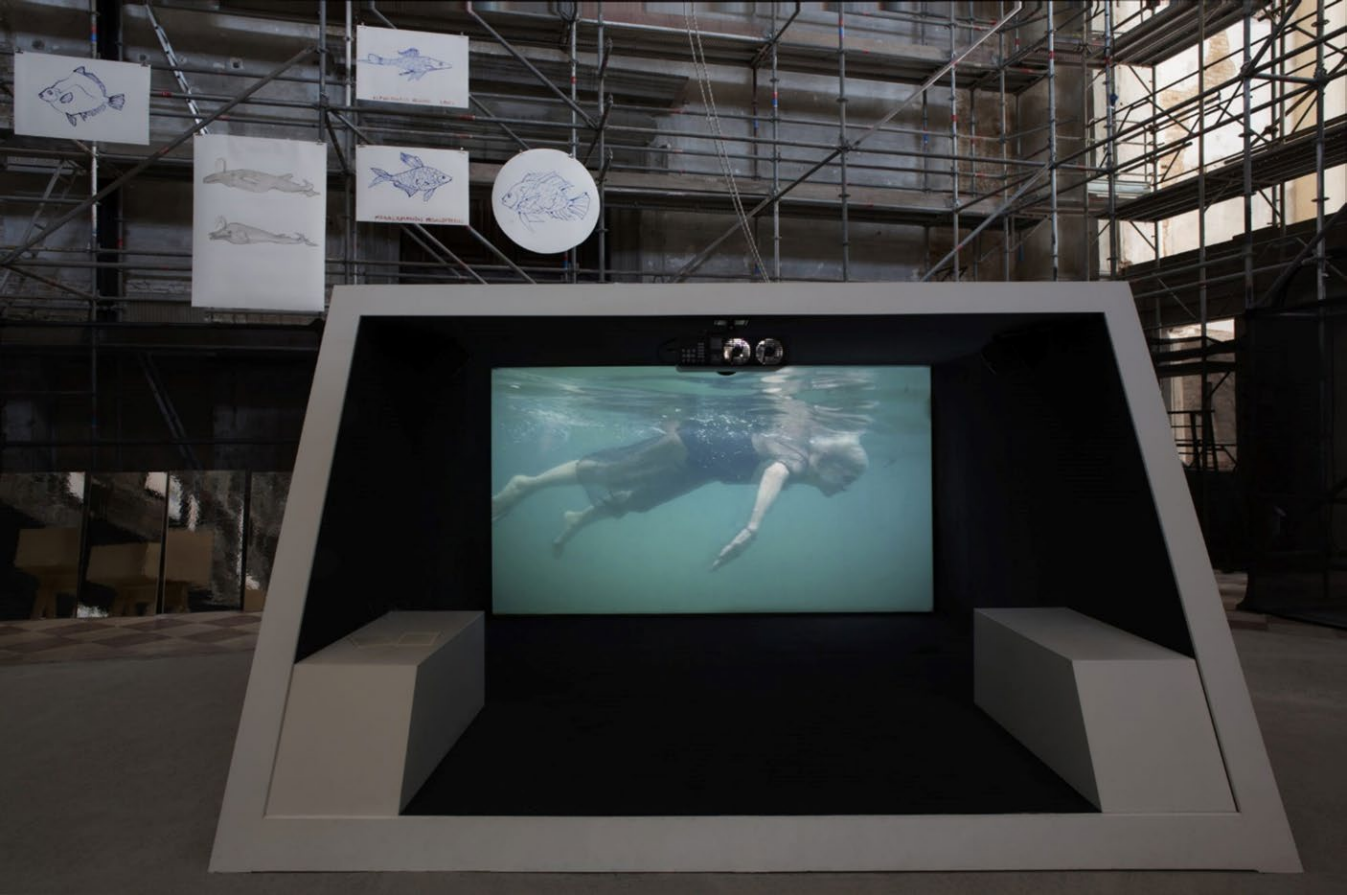
Joan Jonas, *Moving Off the Land II*, at Ocean Space, Church of St. Lorenzo, 2019. *Moving Off the Land II* is commissioned by TBA21–Academy and co-produced with Luma Foundation. Photo Moira Ricci

*Moving Off the Land II* takes the form of a multimedia installation that includes, among other material, drawings and paintings of fish, video projections of various types and formats, and display cases (Fig. 6). As part of the artwork, the artist can occasionally stage performances, an art form she pioneered in the 1970s. The piece is the result of several years of research and residencies. During that time, Jonas took the opportunity to discuss one of her research themes, decentering the human and its perception, with US marine biologist David Gruber^11^. She explains:

“In my own work, there is an underlying focus on how we perceive and how we see what lies before us in our world. In my performances, installations, and single-channel video works, I experiment with materials and simple technologies such as mirrors, distance in landscape, and/or video in order to alter perception and vision. So when I read that David is concerned with how fish perceive, and how he is developing ways for us to experience the phenomenon of biofluorescence in the deep sea, I was immediately drawn to his work” (Jonas in Gruber, 2019).

For his part, Gruber comments, “Experiencing Joan’s work evokes feelings in me that are related to my underwater experiences and deep connection to the submerged community. An Inner Fish” (Gruber, 2019). The installation includes underwater photo and video footage shot by the diver biologist himself. Gruber also studies the communication and language patterns of marine mammals, specifically sperm whales (Bermant et al., 2019)^12^. Recordings of sperm whales made by him and his research team are used as sound material in Jonas’ artwork. The biologist explains his interest in the artist’s work:

“I’m interested in finding ways of altering our own human perspectives. I believe that only by doing so can we learn to cope with the ecological urgencies of our time, and acknowledge the manifold more-than-human perspectives coexisting with us on this blue planet. Joan’s working process, her performance, and exhibition, are powerful attempts to do exactly that” (Gruber, 2019).

**Fig. 7:**
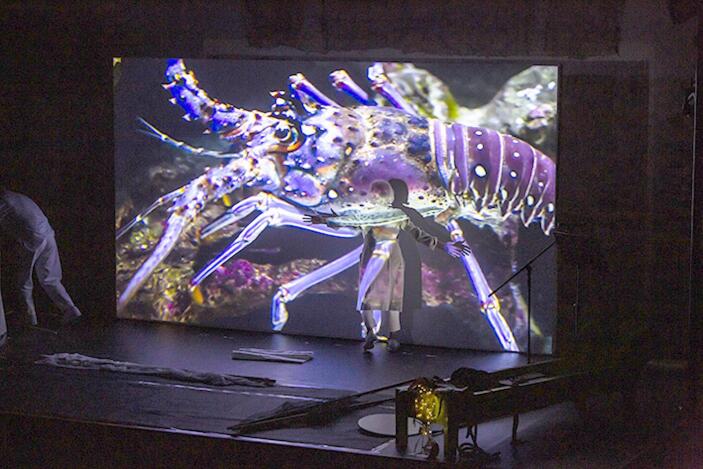
Joan Jonas, *Moving Off the Land II*, performance, Ocean Space, Venice, 2019. Artist interacting with biofluorescent footage by David Gruber

However, this is only one dimension of *Moving Off the Land II*. Through the use of material collected by Gruber and his team, the artist also aims to present a register of different kinds of relationships – in this case scientific and professional – with the ocean and marine animals. In contrast to the images taken by the biologist, she also presents amateur underwater video footage offering an alternative, more popular perspective on this environment. Other images were captured by the artist herself in various aquariums around the world, which she considers as fundamental means of researching on the animals we can observe here without the professional apparatus of scuba diving^13^. During performance, Jonas partially projected these various images – or Gruber’s – onto her body, and simulated interaction with animals, thus metaphorically establishing a seamless connection between their living environment and the human-dominated exhibition/performance space (Fig. 7).

**Fig. 8:**
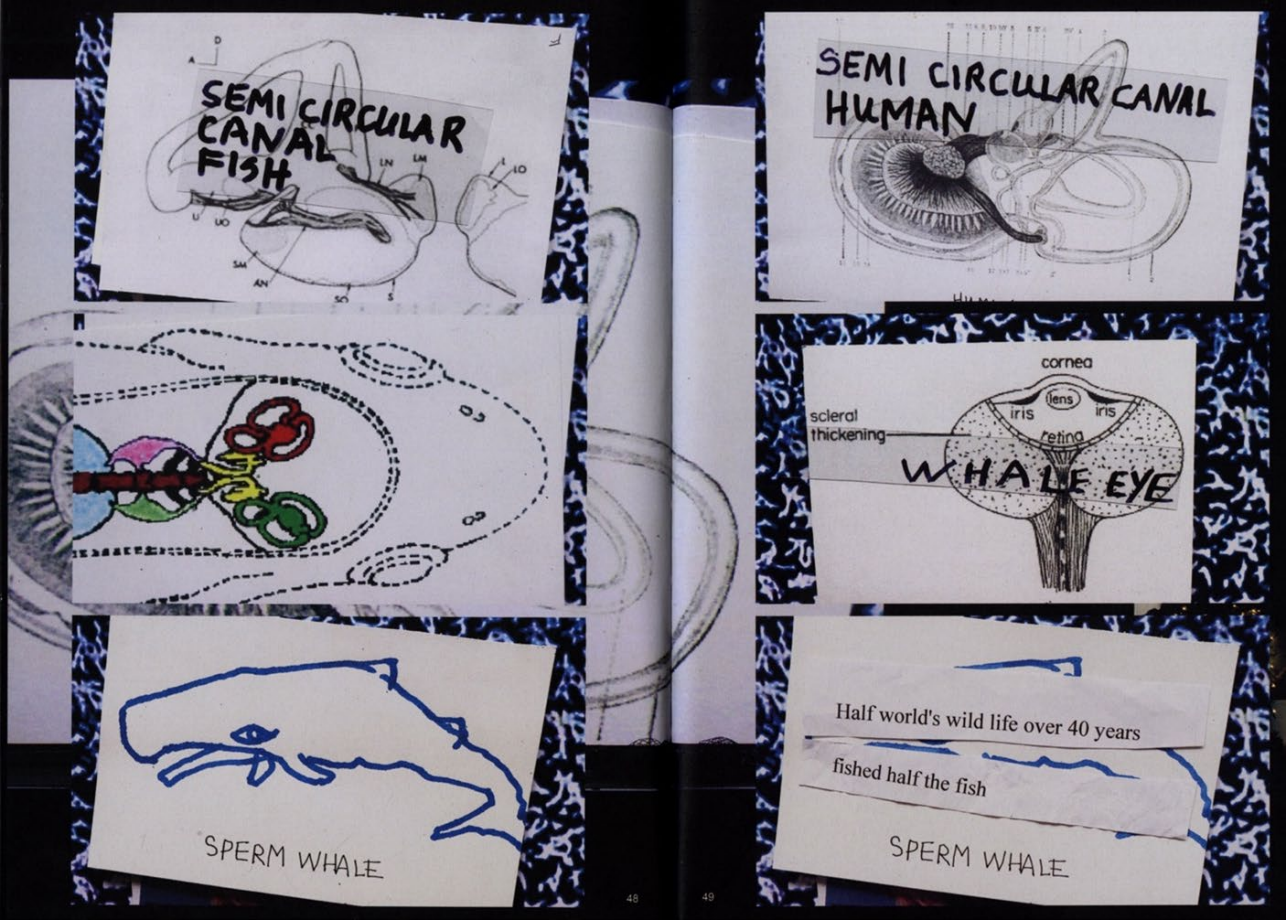
Material used by Joan Jonas in *Moving Off the Land II*, 2019. From Ute Meta Bauer (ed.), *Joan Jonas*,* Moving Off the Land*, Walther König, Köln, 2022

In the piece, the artist also presents medical diagrams that depict the structure of semicircular canals in fish ears, as well as their human counterparts (Fig. 8). She aims to underscore the shared physiological feature between marine and terrestrial species, thereby emphasizing the biological connections between them. Jonas also elaborates on the marine origins of human life. “Reflecting on the reactions from various members of my audience, I am touched that some people don’t realize that we come from the sea and that we are composed of the same elements. Our blood is salty, like salt water” (Jonas in Gruber, 2019). Through various processes, in *Moving Off the Land II*, she seeks to merge scientific knowledge with a mythological register. For instance, some of the video footage features shows the artist herself, swimming in the sea in a dress, her eyes wide open (Fig. 6). The figure of the mermaid plays a key role in the piece, symbolizing the reintegration of humans into their original marine environment. In her performance, the artist also portrays herself as a grotesque and completely unrealistic mermaid, wearing a hat made of seaweed, which adds a significant element of humor to her approach.

**Fig. 9:**
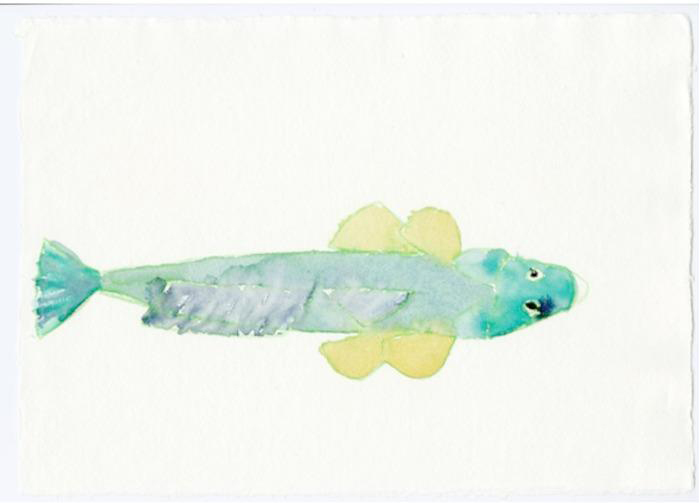
Joan Jonas, *Fish Drawings*,* Moving Off the Land II*, 2019

**Fig. 10:**
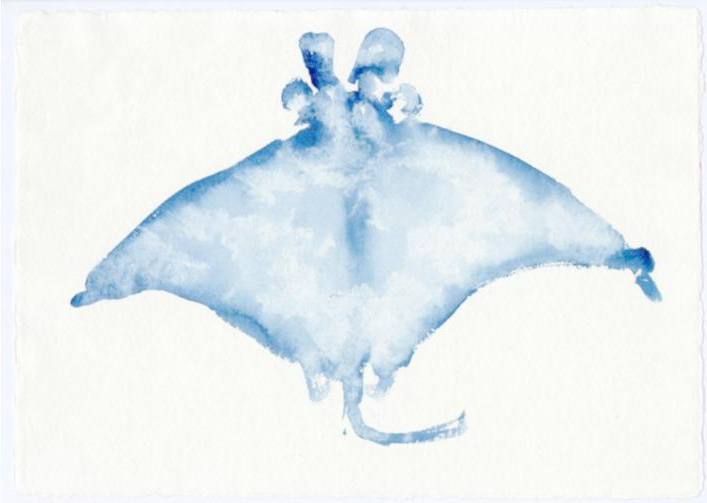
Joan Jonas, *Fish Drawings*,* Moving Off the Land II*, 2019

Among visual material (Figs. 9 and 10), audio excerpts of text are also incorporated into the piece, overlaid on the video extracts and images. Again, these texts come from a variety of registers – scientific, mythological, literature, etc. – all contributing to the transmission of different forms of knowledge about the ocean. This includes, for example, recent texts by the popular naturalist and writer Sy Montgomery, who highlights the specific personalities and particular relationships she developed with octopuses both in aquariums and underwater in Polynesia (Montgomery, 2015). Other earlier extracts borrow from poets Emily Dickinson or Anna Akhmatova, both of whom, in their own unique ways, explored the connections between human beings and the oceanic environment, as well as the philosophical aspects that can deepen human understanding of the ocean. The writings of biologist and writer Rachel Carson, to whom Jonas dedicated *Moving Off the Land II*, particularly exemplifiy a shift away from a standardized scientific framework when engaging with the ocean. Indeed, Carson’s writing style combined science with poetry, and even incorporated a sense of spirituality, as seen in her 1950s bestseller, *The Sea Around Us* (Carson, 1951).

The violent aspect of the historical relationship between humans and the ocean is not absent in *Moving Off the Land II.* The presence of Herman Melville’s novel *Moby Dick* (1851), a fictional account largely informed by scientific knowledge of sperm whales at the time (Hoare, 2013), alludes to the brutality inflicted upon both marine animals and seafarers throughout history. It is also a way for the artist to involve fishermen in her installation, highlighting the unique relationship these humans have developed with the ocean. As she explains, “they depend on the ocean. It is a relationship that we must recognize” (Jonas in Bauer, 2022). During research residencies, Jonas discussed the evolution of the presence of various marine animals in recent decades with local fishermen, particularly in Jamaica, an area that has experienced heavy overfishing but now benefits from conservation measures.

The various texts chosen by the artist are sometimes read by the artist herself, sometimes by children or young people who take them over. By that means, she aims to evoke future scenoarios where where humans are directly connected to the ocean’s ecological state. *Moving Off the Land II* was commissioned by TBA21-Academy, a European-based cultural organization dedicated to the protection of the ocean and addressing related social issues. According to its co-director, Markus Reymann, giving these young people a voice “in a work addressing the massive and rapid changes the world’s oceans are undergoing due to climate emergency, industrial overfishing, and a generally resource-focused relationship to the ocean was very powerful” (Reymann, 2022), especially in the context of 2019, a time when the Youth for Climate movements were simultaneously emerging in Europe.

Although the issue of ocean conservation is present in the discourse surrounding *Moving Off the Land II*, the artist, again, never directly links the ocean to operational concepts such as ES. For her, the motivation for conservation lies elsewhere, in a different way of looking at the ocean and to situate ourselves, as humans, in relation to it. By highlighting a wide range of different registers in our perceptions of and relationships to the ocean, the piece informs us about the historical construction of some human knowledges and sensitivities to it. The artist presents the oceanic environment both as a subject of human history and projection, and as a non-human world, home to animals whose ways of perceiving will always remain beyond our understanding. These two perspectives coexist within the piece. In doing so, it introduces a new dimension to the value that art can assign to the ocean. The artistic value of the ocean conveyed in this case study encompasses a strong societal dimension: art serves to question current social markers, and to potentially establish new ones, that would allow another human relationship to marine species and environment. These questions are highly related to contemporary human and social sciences of the ocean, especially in the field of philosophy of science (for example Haraway, 2008, Alaimo, 2013). Here, art serves as a privileged means of communication with contemporary environmental humanities research, striking a balance between canonical scientific information and various other types of narratives or forms of knowledge related to the ocean. From the artist’s perspective, these should now be activated together to challenge the value we assign to the ocean.

### Critical analysis

Through the artistic values of the ocean that emerge from these three cases, we observe that contemporary artists have numerous ways to actively work towards modifying human perception of the ocean’s known and unknown environments, its ecosystems, marine animals and their life processes. In each case, art plays a crucial role in communicating and advocating various forms of knowledge about the ocean more ore less directly and in raising awareness of conservation policies. Some artists, such as Nicolas Floc’h, can choose to directly integrate their work into management and conservation plans. In such a case, the attention sparked by works of art may facilitate the integration of embodied knowledge (as understood in the philosophy of art) between art and science, potentially driving further implications for ocean conservation – provided that the conservation plans themselves are comprehensive enough. Embodied knowledge is not a literal integration of scientific findings, nor a pedagogical experience, but an artistic experience instilled into the subject (Johnson, 2019) – in this case, the ocean. Irene Kopelman takes a similar approach, but more radically turned away from the ES concept, clearly showing a lack of interest in the characteristics of certain marine species that could be highly valuable into its framework. In her work, artistic creation emerges as a field of activity that, while remaining dependent on other mechanisms of valorization – such as the art market–, claims to distance itself from the traditional ways of valuing the ocean in global North societies, based on a dynamic of resource extraction at various scales. Here as in other cases studied (Anderson et al., 2019), the artistic representation process can provide a fresh approach to scientific exploration, where drawing is regarded as a novel “way of knowing” about the dynamic nature of living systems. The artistic value incorporated into the study of marine life forms thus brings a degree of exploratory imagination to the forefront of scientific practice. The notions through which the work of these two artists operates, namely knowledge and awe, are both considered to be “psychospiritual values” forming part of cultural ES, but specifically straddling instrumental and intrinsic values of the ocean (Schröter et al., 2014). Finally, Joan Jonas attempts to challenge these values from a historical perspective, leading to a more general questioning of how humans relate to the marine environment and species. Her work thus appeals to emotions that are crucial for stimulating conservation motivations beyond the ES paradigm. In particular, interspecific connections are brought to the fore in order to unravel the underlying hierarchy of species. The de-centering of the anthropocentric perspective in her work is not exclusive, but it is radical, especially evident in her exchange with Gruber and their research on non-human perceptions and forms of intelligence or language. Both take some distance from the consideration of human beings at the top of a hierarchy in the biosphere, and of human interests in ocean exploitation as a priority. Through their collaboration on the artist’s work, which involves the use of materials collected by the researcher, they worked together to raise awareness on shared scientific and mythological knowledge, such as the interdependence and continuum between species. As the artist reminds us, “we [humans] actually did come out of the oceans, it is not a myth” (Jonas in Bauer, 2022). The artist’s concept of decentering prompts a reconsideration of the values traditionally associated with the ocean in global North liberal societies, as already theorized in broader works on environmental conservation (Büscher and Robert Fletcher, 2020). With a proposal like Jonas’, it is, undoublty, political orientations conveyed through artistic value. This presents the potential for a shift that could lead to a new paradigm for ocean conservation.

In this critical analysis, our intention is not to radically reject the concept of ES, as we believe it remains a valuable communication tool. However, we believe it can more actively coexist with other modes of sensitivity and intellectual frameworks to refer to the ocean. Through its artistic value, art opens additional avenues for understanding the ocean and positioning our societies in relation to marine life and conservation strategies. Emotions – shaped by bodily engagement and the senses significantly influence how humains relate to the environment and other species. This is the case for scientists as well. Contemporary art delves into these emotional depths in ways which lines up more or less directly with scientific or conservation motivations. Through these examples, we aimed to demonstrate how art fosters a “knowledge culture” other than the one that leads to management in terms of ES alone (Adloff & Hilbrich, 2021).

## Conclusion

Beyond being a platform for sharing scientific research or promoting ocean literacy, as arts & sciences productions are currently too often treated in both ocean policies and interdisciplinary research, these works of art possess unique qualities that should be seriously considered in the broader context of ocean conservation. As demonstrated here, art offers unique means of bringing ocean-related implications and affects to life, both on an individual level and within broader social groups. It does so through plastic and narrative inventiveness combined with the power of imagination. As noted by Professor Kathryn Yusoff, who specializes in Inhuman Geography, the call for imagination has become integral to a new approach in valuing biodiversity:

“Valuing biodiversity […] prompts a call for imagination in how biotic subjects are formed, enacted, practiced, legalized, conducted, regulated and sustained as entities in the cohabitation of the earth. […] Concentrating on the service economy of the biotic world speaks to a limited imagination of what it means to co-inhabit such configurations” (Yusoff, 2011).

Through the impetus of artistic value, the call to imagination extends beyond artists and the general public – including those distant from the ocean – to decision-makers primarily engaged with the ES framework. The latter is based on transforming negative values associated with the logic of conservation policies, such as concerns over extinction, into positive ones, like services to humans (Brunet, 2022). Beyond the specific cases presented here, artistic value can deeply engage decision-makers, but also philosophers, and their communities, by concretizing both the limitations of purely utilitarian approaches – those based on ES – and purely deontological ones, focused on intrinsic values. In this way, artistic value helps move beyond dualism and should be considered as a potential source for well-informed decision-making in ocean conservation, playing a significant role in how our societies engage with the ocean. Recognizing shared or contrasting approaches – without opposing them – can shape effective conservation strategies and, if expanded, offer a new paradigm for promoting ocean conservation. The growing importance of emerging oceanic narratives and their visual representations are now considered crucial in shaping future views of the ocean, which remains largely unfamiliar to us and will continue be. This aligns with broader shifts in political understanding of the ocean.

## Footnotes

1 In the ocean, they are mainly defined as biological and mineral resources, harvested to provide food, energy and medicine.

2 The best example on land is pollination, a service that would not be possible without insects. In the ocean, climate regulation is widely acknowledged as the most important service, the main debate today being to assess the extent to which marine ecosystems can cope with and/or mitigate climate change.

3 This type of ES includes organism physiological mechanisms, such as photosynthesis, which is at the basis of most trophic networks, and also extend to the genetic level, as genetic diversity can indicate the health status of a population. For example, the value of pollination to our economy has been estimated (Porto et al., 2020), but no one can say what the value of photosynthesis is, or what is at stake for humankind after recurrent loss of genetic biodiversity due to the extinction of local populations. What kind of economic compensation can be proposed for the loss of such ES?

4 This research should take into account the various challenges already identified in the case of cultural ES (Gould et al., 2020).

5 This concept differs from the sense of embodied knowledge as ‘know-how’, i.e. practical knowledge, as usually understood in the philosophy of science as a complement to theoretical knowledge (‘knowing that’). (Leonelli, 2009, p. 7). However, the two concepts overlap to some extent, as will be explained later.

6 All translations are by the authors.

7 (CNRS, IRD, INRAE). Together with Fondation Camargo.

8 See for example the *Science-Art Initium Maris* project conducted by Floc’h with marine ecologist Sylvain Agostini (SMRC Laboratory of Marine Eco-Physiology) on the tropicalization of marine ecosystems off Brittany and Japan. In this context, the transformation of underwater seascapes is directly examined from the perspective of the modification of the “ecosystem services they provide” (Thalie Foundation, 2021). Here, art as a proposition for ‘embodied knowledge’ seems to be integrated with no difficulties into the canonical ES framework.

9 Residency supported by IDEX UCAjedi, in preparation for an exhibition at MAMAC. https://univ-cotedazur.eu/research-program#image.

10 Earlier versions of the piece were performed between 2016 and 2018, notably at Fort Kochi, New York, San Francisco, and London, under the title *Moving off the Land*. *Moving off the Land II* is a work that can evolve with each new installation, as demonstrated at the Museo Nacional del Prado in Madrid in 2020. Performance available at: https://www.youtube.com/watch?v=jzQiyoO_qcs.

11 Professor of biology and environmental sciences at Baruch College and the Graduate Center, CUNY.

12 Gruber is also the founder of the Founder of Project CETI (Cetacean Translation Initiative), a scientific and conservation initiative studying the communication of sperm whales.

13 Footages shot in the Lofoten Aquarium, the New England Aquarium, the New York Aquarium at Coney Island, NY, the National Aquarium in Baltimore, MD., the Genoa Aquarium, the Mystic Aquarium in Mystic, CT, and various Aquariums in Tokyo.
